# Photoacoustic and Fluorescence Imaging of Cutaneous Squamous Cell Carcinoma in Living Subjects Using a Probe Targeting Integrin α_v_β_6_

**DOI:** 10.1038/srep42442

**Published:** 2017-02-09

**Authors:** Chao Zhang, Yong Zhang, Kai Hong, Shu Zhu, Jie Wan

**Affiliations:** 1 Department of Medical Ultrasound, Tongji Hospital, Tongji Medical College, Huazhong University of Science and Technology, Wuhan, 430030, China; 2 Department of Dermatology, Tongji Hospital, Tongji Medical College, Huazhong University of Science and Technology, Wuhan, 430030, China; 3 Department of Pathology, Tongji Hospital, Tongji Medical College, Huazhong University of Science and Technology, Wuhan, 430030, China

## Abstract

Cutaneous squamous cell carcinoma (cSCC) is the second most common non-melanoma skin cancer worldwide. Today, cSCC is diagnosed by visual inspection followed by invasive skin biopsy. There is a need to develop non-invasive diagnostic tools to achieve early and accurate detection. Photoacoustic imaging (PAI) possesses high ultrasonic resolution and strong optical contrast at new depths (<1–5 cm). Together with exogenous contrast agents, PAI has found promising use in various tumors in living subjects. The expression of integrin α_v_β_6_ is significantly up-regulated in cSCC. We fabricated an anti-integrin α_v_β_6_ antibody and labeled it with indocyanine green (ICG) to form an ICG-α_v_β_6_ antibody. The results showed that the ICG-α_v_β_6_ antibody probe could be used to detect cSCC with high specificity (3-fold over the control by PAI) and deep penetration (approximately 1 cm) by PAI. This suggests that the ICG-α_v_β_6_ antibody is a promising probe targeting the integrin α_v_β_6_ for detection of cSCC tumors by PAI and fluorescence imaging. It may find clinical application in the early diagnosis of cSCC as well as in intraoperative navigation.

Non-melanoma skin cancer (NMSC) is the most common cancer worldwide. While the majority of NMSCs are in the form of basal cell carcinoma, cutaneous squamous cell carcinoma (cSCC) is the second most common pathology, accounting for 20% of all cutaneous malignancies^1^. cSCC can metastasize unless treated early by optimal surgical techniques, and thus early diagnosis is important. Today, cSCC is diagnosed by visual inspection followed by invasive skin biopsy^2^. There exists a need to develop non-invasive diagnostic tools to achieve early and accurate detection.

Dermoscopy, reflectance confocal microscopy and optical coherence tomography are being used as diagnostic tools prior to surgery^3^. However, all of these optical devices have limited detection depth (<1 mm)^2^. High-frequency ultrasonography can give a clear picture of the size and depth of the tumor but is not suitable for differential diagnosis^3^. Recently, photoacoustic imaging (PAI) was developed as an imaging technology based on the photoacoustic effect. In PAI, pulsed light energy is converted to heat after being absorbed by an endogenous absorber (e.g., melanin or hemoglobin) or exogenous absorber (e.g., dyes or nanoparticles). The heat causes the absorber to undergo rapid thermoelastic expansion and generates an ultrasound wave that can be detected with a conventional ultrasound transducer^4^. PAI possesses high ultrasonic resolution and strong optical contrast in optically scattering biological tissue at new depths (<1–5 cm)^5^. Together with exogenous contrast agents, PAI has found promising use in various tumors in living subjects.

There are a variety of imaging agents, including organic dyes, nanoparticles and reporter genes, which can be used for PAI. The advantages of using small-molecule fluorescent dyes for *in vivo* imaging are their biocompatibility and rapid clearance from the body. In addition to a few of these imaging dyes that have been approved for human use, the rest of the imaging agents are not yet approved. In this study, we selected a near-infrared fluorescent dye, indocyanine green (ICG), to serve as a multimodal fluorescence and photoacoustic contrast agent. ICG has been in clinical use for decades for retinal angiography and liver function studies. Recently, it is expected to achieve sensitive fluorescence and photoacoustic signals; having been developed for tumor imaging, it is potentially applicable in clinical photoacoustic imaging^6^.

Integrins are a family of heterodimeric cell surface receptors. Integrin α_v_β_3_ has been shown to be expressed on the surface of cancer cells and the tumor neovasculature. However, in certain cancers, integrin α_v_β_6_ becomes highly overexpressed on cell surfaces and is undetectable in most normal adult tissues^7^. The expression of α_v_β_6_ is significantly up-regulated in cSCC^8^,^9^.

In our previous study^10^, the probe A740-R_0_1, a peptide labeled with the fluorescent dye Atto 740, was able to detect α_v_β_6_-positive tumors in living subjects, but the signals were relatively weak. Moreover, Atto 740 is still not approved for human use.

In this study, we plan to fabricate an anti-α_v_β_6_ antibody, label it with ICG and evaluate the ability of the ICG labeled antibody to detect cSCC tumors by PAI and fluorescence imaging.

## Results

### Production of anti-α_v_β_6_ antibody

**Figure 1:**
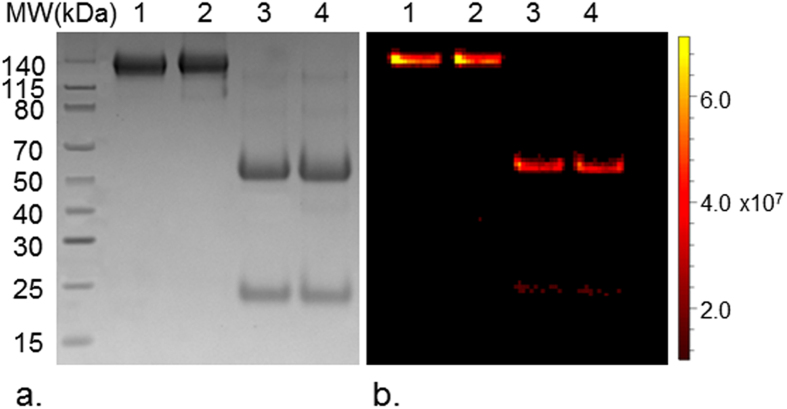
Biochemical analysis of self-made antibody. (**a**) Non-reducing (lanes 1 and 2, exempted from 2-mercaptoethenol and heating) and reducing (lanes 3 and 4) SDS-PAGE of the affinity purified anti-α_v_β_6_ antibody (lanes 1 and 3) and scrambled IgG (lanes 2 and 4) produced from 293F cells transiently transfected with pFUSE_H and pFUSE_L. (**b**) Anti-α_v_β_6_ antibody and scrambled IgG were labeled with ICG. Fluorescence imaging of the SDS-PAGE of ICG-α_v_β_6_ antibody and ICG-ScrIgG validated the covalent conjugation of ICG to antibody. Full length gel images are presented in Supplementary Fig. S2.

Expression yields for anti-α_v_β_6_ antibody from FreeStyle 293 F cells were approximately 33.2 mg/L (Supplementary Fig. S1) following NAb Protein G Spin column purification. Under non-reducing conditions, SDS-PAGE revealed a single band present at ~150 kDa. Reducing SDS-PAGE conditions for the antibody revealed two bands at ~51 kDa and 23 kDa, representing the heavy chain and light chain of the antibody (Fig. 1a, Supplementary Fig. S2a). Mass spectrometry further confirmed the molecular weight of the antibody. A clear peak corresponding to the entire antibody was visible at 150,319 m/z (Supplementary Fig. S3a). Under reducing conditions, peaks of the heavy chain and light chain were visible at 52,609 m/z and 22,419 m/z (Supplementary Fig. S3b).

### Synthesis and characterization of ICG-conjugated anti-α_v_β_6_ antibody

The covalently bound ICG on the ICG-α_v_β_6_ antibody and ICG-ScrIgG could be observed on reducing and non-reducing SDS-PAGE by the fluorescence imaging system (Fig. 1b, Supplementary Fig. S2b). The conjugation ratio of dye to antibody was 5.1 and 5.2 for ICG-α_v_β_6_ antibody and ICG-ScrIgG, respectively, as calculated from the optical spectrum (Supplementary Fig. S4).

**Figure 2:**
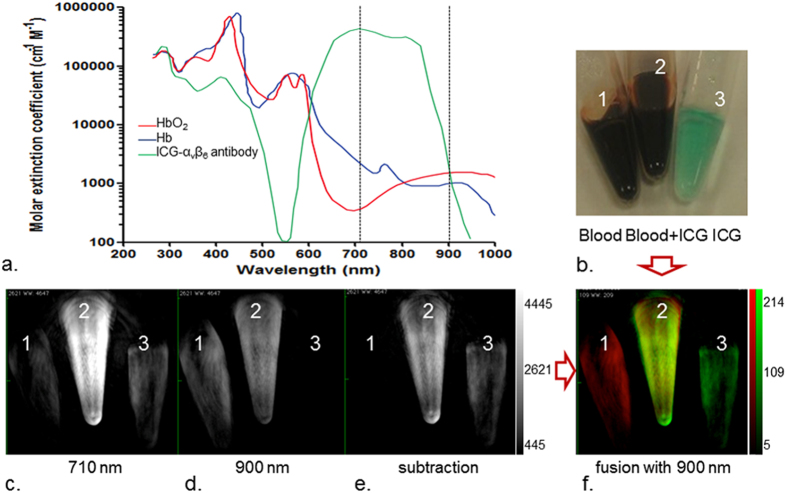
Optical and photoacoustic characterization of the ICG-α_v_β_6_ antibody. (**a**) Molar extinction spectra of oxygenated hemoglobin (HbO2, red line), deoxyhemoglobin (Hb, blue line) and ICG-α_v_β_6_ antibody (green line). There exists a dramatic difference in the extinction coefficient of ICG-α_v_β6 antibody at 710 nm and 900 nm. Limited difference exists for HbO_2_ or Hb at 710 nm and 900 nm. (**b**) Venous blood of mice in tube 1, mixture of blood and ICG-α_v_β_6_ antibody in tube 2, and ICG-α_v_β_6_ antibody in tube 3 were arranged on the animal tray of the photoacoustic imaging (PAI) instrument. (**c**) PAI at 710 nm showed the photoacoustic signals from all three tubes. (**d**) Only signals from blood were visible at 900 nm. No obvious photoacoustic signal could be observed from the ICG-α_v_β_6_ antibody in tube 3. (**e**) Subtraction of images at 710 nm and 900 nm shows the photoacoustic signal from ICG-α_v_β_6_ antibody in tube 2 and tube 3. Signals from blood were subtracted. (**f**) The photoacoustic signals from blood (red) and ICG-α_v_β_6_ antibody (green) were un-mixed well by subtraction and fusion of images at 710 nm and 900 nm.

The maximum absorption wavelength of the ICG-α_v_β_6_ antibody was 710 nm (Fig. 2a, Supplementary Fig. S4). The strongest signal from the ICG-α_v_β_6_ antibody can be detected by photoacoustic instrument at laser light 710 nm, while a limited photoacoustic signal could be detected at 900 nm. On the other hand, for mouse blood, photoacoustic signals at 710 nm were a little bit weaker than those at 900 nm. Subtraction of the images obtained at 710 nm and 900 nm could eliminate the signals from blood and highlight the signals from the ICG-α_v_β_6_ antibody (Fig. 2b,c).

No significant decrease of photoacoustic signal from the ICG-α_v_β_6_ antibody was observed after irradiation with 710 nm and 900 nm laser light for 30 min.

### Binding affinity

**Figure 3:**
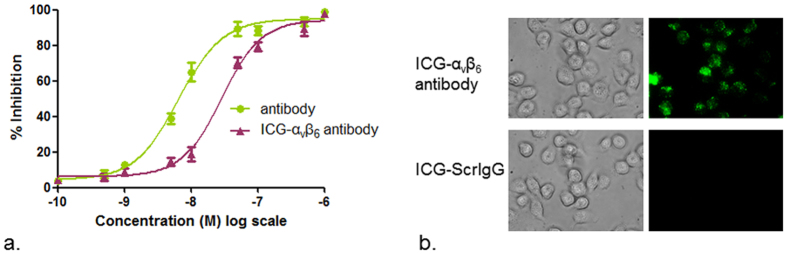
Binding affinity study. (**a**) Determination of binding affinity by competitive binding ELISA for the anti-α_v_β_6_ antibody (green, IC_50_ = 6.5 nM) or ICG-α_v_β_6_ antibody (brownish red, IC_50_ = 28.4 nM) following incubation with fibronectin against immobilized integrin α_v_β_6_. Each data point represents the mean of triplicate experiments and errors bars indicate standard deviation. (**b**) Microscopic fluorescence images of cSCC A431 cells (integrin α_v_β_6_ positive) incubated with ICG-α_v_β_6_ antibody or ICG-ScrIgG for 4 h at 37 °C. Higher signals were observed after incubation with ICG-α_v_β_6_ antibody.

The binding affinity of the anti-α_v_β_6_ antibody and ICG-α_v_β_6_ antibody against immobilized integrin α_v_β_6_ (n = 3 each) was demonstrated (Fig. 3a). Sigmoidal curves were fitted to the absorbance data using the software GraphPad Prism (GraphPad, La Jolla, CA). The IC_50_ values of the anti-α_v_β_6_ antibody and ICG-α_v_β_6_ antibody towards integrin α_v_β_6_ were 6.5 nM and 28.4 nM, respectively. Competitive binding ELISAs against integrin α_v_β_3_ and α_v_β_5_ were used to confirm the specificity of the antibody towards α_v_β_6_ (Supplementary Fig. S5a,b).

In microscopic study, higher fluorescence signals within integrin α_v_β_6_ positive A431 cells could be observed after the cells were incubated with ICG-α_v_β_6_ antibody than with ICG-ScrIgG (Fig. 3b). Much lower fluorescence signals within A431 cells were observed when they were blocked with excess unlabeled antibody before the addition of the ICG-α_v_β_6_ antibody (Supplementary Fig. S5c). A limited fluorescence signal could be observed within integrin α_v_β_6_ negative 293 T cells (Supplementary Fig. S5d). These results confirm the specific binding of the ICG-α_v_β_6_ antibody to integrin α_v_β_6_-expressing cells.

### Sensitivity Study

**Figure 4:**
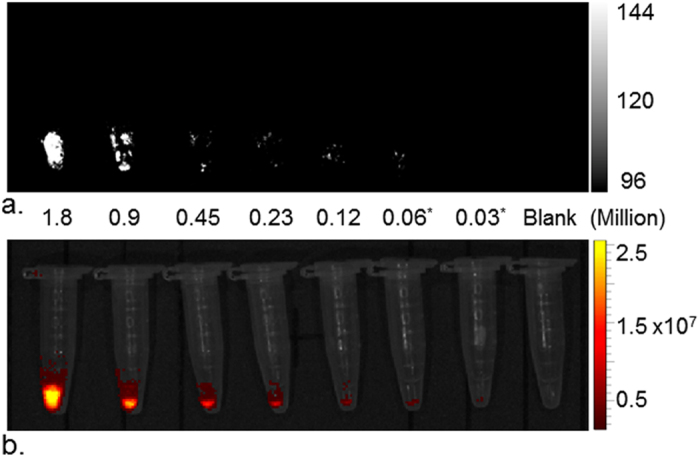
Sensitivity study. (**a**) Photoacoustic and (**b**) fluorescence imaging of various numbers of cSCC A431 cells labeled with ICG-α_v_β_6_ antibody. The lowest detectable number of labeled A431 cells was 0.06 million by photoacoustic imaging and 0.03 million by fluorescence imaging.

The minimum detectable number of A431 cells incubated with ICG-α_v_β_6_ antibody was determined to be 0.06 million by PAI (*P* = 0.03 when compared with blank) and 0.03 million by fluorescence imaging (*P* = 0.04 when compared with blank) (Fig. 4).

### Small Animal Imaging and Tissue Biodistribution

**Figure 5:**
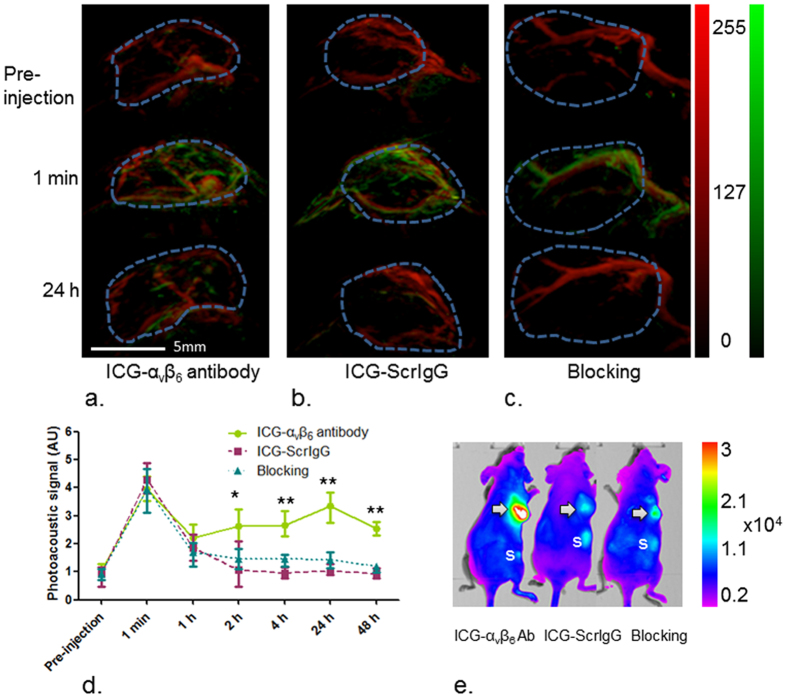
*In vivo* imaging of tumors. (**a**,**b**,**c**) cSCC xenograft tumors in 8-week female nude mice (nu/nu, n = 9) were imaged at 710 nm and 900 nm by PAI. The images at 710 nm were subtracted by images at 900 nm and then fused with images at 900 nm. The un-mixed signals from blood (red) and probe (ICG-α_v_β_6_ antibody or ICG-ScrIgG, green) were then presented simultaneously. Transient enhancement of photoacoustic signal 1 min after tail vein injection of probe demonstrated the arterial phase of the probe in blood circulation. (**a**) Obvious enhancement of photoacoustic signal could be observed 24 h after tail vein injection of the targeted ICG-α_v_β_6_ antibody, (**b**) while there was only a slight change after injection of non-targeted ICG-ScrIgG. (**c**) The enhancement of signal was inhibited when blocked with unlabeled anti-α_v_β_6_ antibody 10 min before administration of ICG-α_v_β_6_ antibody. (**d**) Higher photoacoustic signals were detected 2 h, 4 h, 24 h and 48 h after injection of ICG-α_v_β_6_ antibody than injection of ICG-ScrIgG and blocking with unlabeled anti-α_v_β_6_ antibody. Maximum enhancement could be observed at 24 h post-injection of ICG-α_v_β_6_ antibody. *P < 0.05; **P < 0.01. (**e**) Fluorescence imaging of xenografts 24 h post-injection of probe. The highest fluorescent signal from cSCC xenografts (Arrow) was acquired after injection of ICG-α_v_β_6_ antibody instead of injection of ICG-ScrIgG and blocking with unlabeled anti-α_v_β_6_ antibody. S, spleen.

The efficacy of the ICG-α_v_β_6_ antibody at detecting cSCC tumors *in vivo* was studied in nude mice (Fig. 5, Supplementary Fig. S6). All photoacoustic images were collected at 710 nm and 900 nm. The images were subtracted to un-mix the probe signals (Supplementary Fig. S6). The un-mixed signals were then fused with images at 900 nm to demonstrate the vasculature of the tumor and signals of the probes simultaneously (Fig. 5a,b,c).

The photoacoustic signals increased dramatically 1 min post injection of probes and then decreased until the 1 h time point. No significant difference of photoacoustic signal could be observed after tail vein injection of ICG-α_v_β_6_ antibody (3.96 AU ± 0.44) and ICG-ScrIgG (4.29 AU ± 0.55; *P* > 0.05) at the 1 min time point or at the 1 h time point (2.22 AU ± 0.43 and 1.86 AU ± 0.47, respectively; *P* > 0.05). Higher photoacoustic and fluorescence signals were observed at all the other time points (2 h, 4 h, 24 h and 48 h) post injection of ICG-α_v_β_6_ antibody compared to ICG-ScrIgG (*P* < 0.05) (Fig. 5d). *In vivo* photoacoustic imaging of cSCC tumors showed a 3-fold higher signal (3.36 AU ± 0.56) 24 h post injection of ICG-α_v_β_6_ antibody than that of ICG-ScrIgG (1.03 AU ± 0.21; *P* = 0.002). The signals could be inhibited by administration of an excess of unlabeled anti-α_v_β_6_ antibody 10 min before injection of ICG-α_v_β_6_ antibody (1.43 AU ± 0.29; *P* = 0.006).

The fluorescence signal from cSCC tumors 24 h post injection of ICG-α_v_β_6_ antibody was 62.96 AU ± 10.99, a 6-fold increase over the value with ICG-ScrIgG enhancement (10.01 AU ± 1.02, *P* = 0.001) and a 4-fold increase over that with blocking antibody added (15.04 AU ± 1.52, *P* = 0.001) (Fig. 5e).

**Figure 6:**
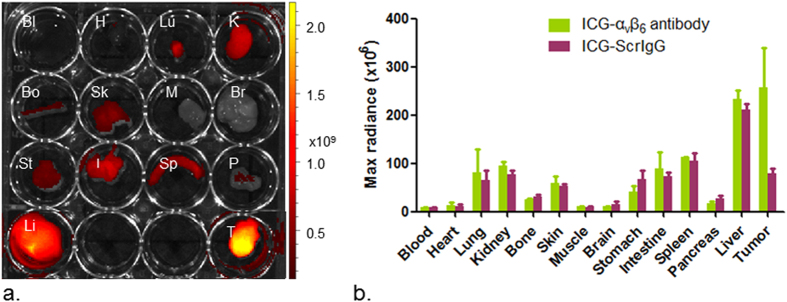
Biodistribution of ICG-α_v_β_6_ antibody. (**a**) *Ex vivo* fluorescence imaging of various organs 48 h after tail vein injection of ICG-α_v_β_6_ antibody. Bl-Blood, H-Heart, Lu-Lung, K-Kidney, Bo-Bone, Sk-Skin, M-Muscle, Br-Brain, St-Stomach, I-Intestine, Sp-Spleen, P-Pancreas, Li-Liver, T-Tumor. (**b**) Higher maximum radiance in tumors was observed than in the non-targeted ICG-ScrIgG control group. No significant difference between the two groups was observed among other organs. Higher maximum radiance was observed in liver, followed by spleen, intestine and kidney.

The biodistribution of probes in various organs and tumors was evaluated *ex vivo* by fluorescence imaging (Fig. 6a). Higher maximum radiance in cSCC tumors was observed 48 h post injection when ICG-α_v_β_6_ antibody was injected compared to ICG-ScrIgG. No significant difference was observed in other organs between the two groups. Higher maximum radiance was observed in the liver, followed by the spleen, intestine and kidney (Fig. 6b) in both groups of mice. Microscope images confirmed the presence of signals from the probe in the tumor tissue of the ICG-α_v_β_6_ antibody injected mouse but not in that of the ICG-ScrIgG injected mouse (Supplementary Fig. S7).

## Discussion

The IC_50_ values of the fabricated antibody and ICG labeled antibody towards integrin α_v_β_6_ were 6.5 nM and 28.4 nM, respectively. The almost 5-fold decrease of IC_50_ value may be caused by the combination of ICG on lysine residues present within the variable domain of HC. This combination can potentially have adverse effects on antigen recognition. Decreasing the labeling ratio of ICG or site-specific binding of ICG may reduce this impact. The low inhibition rate of the fabricated antibody against integrin α_v_β_3_ and α_v_β_5_ in competitive binding ELISAs confirms the specificity of the antibody towards integrin α_v_β_6._ The lower fluorescence signal within A431 cells (α_v_β_6_ positive) in the blocking study and limited signals within 293 T cells (α_v_β_6_ negative) further confirm the specific binding of the ICG-α_v_β_6_ antibody to integrin α_v_β_6_.

Our *in vitro* study also showed that 0.03 million cells can be detected by fluorescence imaging. If 10 million cells are assumed to be in a 100 mm^3^ tumor^11^, the ICG-α_v_β_6_ antibody could theoretically detect lesions as small as 0.3 mm^3^ by fluorescence imaging. *In vivo* study showed xenograft cSCC tumors to be clearly visible by PAI and fluorescence imaging post injection of ICG-α_v_β_6_ antibody. All these indicate that ICG-α_v_β_6_ antibody is potentially useful in diagnosis of cSCC, including special types of SCC such as subungual squamous cell carcinoma^12^, because of the deep detection ability of PAI. Moreover, because high levels of integrin α_v_β_6_ expression have been found at the invasive edge of carcinomas^13^, the ICG-α_v_β_6_ antibody may increase the ability to obtain negative resection margins and visualize residual tumor during surgery by PAI and fluorescence imaging.

A dramatically increased photoacoustic signal 1 min after injection of probes demonstrates the arterial phase of the probes in blood circulation. The similar level of photoacoustic signal enhancement confirms that the same dose of probes (targeted ICG-α_v_β_6_ antibody or untargeted ICG-ScrIgG) was injected into the subjects. A higher signal at other time points after injection with ICG-α_v_β_6_ antibody compared to the ICG-ScrIgG and blocking groups confirms the targeted specificity of the ICG-α_v_β_6_ antibody against tumor.

In a previous study^10^, a knot peptide of approximately 3.8 kDa was labeled with another organic dye, Atto 740, for targeted photoacoustic imaging of tumors *in vivo*. A 2-fold increase over the value before injection was observed. The ICG labeled antibody fabricated in this study with 150 kDa molecular weight achieved a 3-fold increase in photoacoustic signal. There exists similar binding affinity between the ICG-α_v_β_6_ antibody (28.4 nM) and the labeled peptide A740-R_0_1 (39.4 nM). The relatively small size of the peptide and the resulting short circulation time in living bodies limits the signal enhancement. The slow-clearing antibody with long half-life exhibits high tumor accumulation^14^,^15^. This enables the ICG-α_v_β_6_ antibody to be a robust probe for targeted imaging of integrin α_v_β_6_ positive cSCC tumors by PAI and fluorescence imaging with high specificity.

There exists a difference between the signal enhancement rate by fluorescence imaging and PAI. Six-fold enhancement was achieved 24 h post-injection of the ICG-α_v_β_6_ antibody in fluorescence imaging. In contrast, PAI showed a 3-fold change. This phenomenon may be attributed to the difference in the efficiency of energy transfer through living tissue via the different modalities and is consistent with previous research^10^.

ICG was selected in this study to label the antibody. Photobleaching may occur after ICG is exposed to light for 1 day^11^. On the other hand, under the irradiation of laser light at 710 nm and 900 nm for 30 min, no significant decrease of photoacoustic signal was observed. This indicates that ICG is relatively stable under certain scanning situations. The ICG labeled ICG-α_v_β_6_ antibody probe will be prone to clinical transformation.

PAI with ICG-α_v_β_6_ antibody allows for targeted imaging of cSCC tumors deep in tissues with high spatial resolution; however, high background signal from blood can limit the achievable detection sensitivity^16^. There are a variety of spectral processing methods to un-mix the signal of probe from blood^17^. In this study, a high photoacoustic signal at 710 nm and a very weak signal at 900 nm could be detected from the ICG-α_v_β_6_ antibody. On the other hand, a relatively weak photoacoustic signal was detected from hemoglobin at both of these two wavelengths. A simple subtraction of images at these two different wavelengths can eliminate the signal from hemoglobin and thereby highlight the signal from the ICG-α_v_β_6_ antibody^18^.

Several limitations of our study need to be addressed. Although un-mixing the signal of ICG-α_v_β_6_ antibody by subtraction of 710 nm and 900 nm images may restrain blood signals, signals from other components, such as melanin and lipids, may not be totally restrained. We will attempt more sophisticated algorithmic analysis for the extraction of signal of the probe from tissues. The random conjugation of ICG to antibody may lead to a decrease of binding affinity of the ICG-α_v_β_6_ antibody for integrin α_v_β_6_. Site-specific conjugation will be considered in future studies. An ultraviolet-induced early cSCC model in mice that has biologic character more similar to human cSCC will also be used. The efficacy and correlations between antibody lots should be assessed particularly before clinical application.

Our results suggest that ICG-α_v_β_6_ antibody is a promising probe targeting the integrin α_v_β_6_ for the detection of cSCC tumors by PAI and fluorescence imaging. It may find clinical application in diagnosis of cSCC as well as in intraoperative navigation.

## Methods

### Production of anti-α_v_β_6_ antibody

The gene segments of the heavy-chain (HC) and light-chain (LC) encoding a derivative motif^10^ targeting integrin α_v_β_6_ were identified after bio-panning for phages in the phage display system^19^. The HC sequence was then inserted into the vector pFUSEss_CHIg_hG1 (Invivogen, San Diego, CA) between EcoRI and NheI restriction sites to form plasmid pFUSE_H. The LC sequence was inserted into the multiple cloning site of the vector pFUSE2ss-CLIg-hl2 (Invivogen) between EcoRI and AvrII restriction sites to form plasmid pFUSE_L.

The plasmids were used to transform E. coli competent cells. Zeocin (for HC) or blasticidin (for LC) resistant clones were cultured and plasmids isolated using a maxi kit (Sigma Aldrich, St Louis, MO). DNA sequencing was performed to confirm the correct sequence.

A scrambled gene sequence was inserted into the variable domain of HC for production of a scrambled IgG (ScrIgG) without specificity for α_v_β_6_.

The antibody was biosynthesized using FreeStyle™ 293 Expression System (Thermo Fisher Scientific, Pittsburgh, PA). FreeStyle 293F cells were grown in DMEM supplemented with 10% FBS and penicillin/streptomycin (Thermo Fisher Scientific) to 90% confluence in plates. Cells were washed and then digested with 2 ml trypsin. The suspended cells were centrifuged (1500 r.p.m, 5 min) to pellets and then resuspended with 10 ml of serum free FreeStyle™ 293 expression medium. Cell concentrations were calculated with a Countess cell counter (Thermo Fisher Scientific). Cell viability should be greater than 90%. Cells were then separated into 125-ml square flasks with final viable cell concentration of 1 × 10^6^/ml and volume of 28 ml in each flask for transfection. Sixty microliters of lipofectamine (Thermo Fisher Scientific) was mixed gently with Opti-MEM^®^ I to a total volume of 1 ml and incubated for 5 min at room temperature. Twelve micrograms of pFUSE_H and 18 μg of pFUSE_L were mixed gently with Opti-MEM^®^ I to a total volume of 1 ml in a 2-ml Eppendorf tube. Then, the diluted lipofectamine was mixed into the plasmids gently and incubated for 30 min at room temperature. After the incubation, the 2 ml of lipofectamine-plasmid complex was added to the prepared flask of cells. Each flask should contain a total volume of 30 ml, with a final cell density of approximately 1 × 10^6^ viable cells/ml. The cells were then incubated in a 37 °C incubator with a humidified atmosphere of 8% CO_2_ in air. At 24 hours after transfection, valproic acid sodium salt (Sigma Aldrich, St. Louis, MO) was added into the cultures with final concentration of 2 mM. Seven days after transient transfection, the culture medium was harvested and centrifuged at 1500 r.p.m. for 5 min. The supernatant was purified with a NAb Protein G Spin Kit (Thermo Fisher Scientific). Sufficient antibody for the experiment was harvested at one time to maintain the same efficacy of the antibody. This means approximately 30 flasks of 293F cells (with 30 ml expression medium in each flask) were transfected simultaneously.

The quality of the preparations was analyzed on reducing and non-reducing SDS-PAGE. Molecular masses were confirmed by matrix-assisted laser desorption/ionization time-of-flight mass spectrometry (MALDI-TOF MS; Foster City, CA). The eluents were condensed and the solvent was changed to 0.1 M NaHCO_3_ (pH = 7.3) using Vivaspin 500 50 kDa MWCO spin membrane (Sartorius Biotech, Goettingen, Germany) and stored at 4 °C or −20 °C for short-term (<1 month) or long-term storage. Concentration of the purified antibody was detected with a Nanodrop 2000 UV-Vis spectrophotometer (Thermo Fisher Scientific).

ScrIgG was also manufactured at the same time in the same way.

### Synthesis and characterization of ICG-conjugated anti-α_v_β_6_ antibody

The solution of anti-α_v_β_6_ antibody (1 mg/mL) in 0.1 M NaHCO_3_ (pH 8.5) was incubated with fluorescent ICG-NHS dye (Intrace, Lausanne, Switzerland) dissolved in anhydrous dimethylformamide (DMF; Sigma Aldrich) in a 1:10 molar ratio for 2 h at 37 °C. After incubation, the ICG-conjugated anti-α_v_β_6_ antibody (ICG-α_v_β_6_ antibody) was purified by Zeba spin column (Pierce Biotechnology, Rockford, IL) and stored in DPBS at 4 °C. The number of fluorochromes per antibody was determined by spectrophotometric analysis (Nanodrop 2000; Thermo Fisher Scientific). The ICG-α_v_β_6_ antibody was analyzed on reducing and non-reducing SDS-PAGE first and then imaged by fluorescence imaging system (IVIS200; Caliper Life Sciences, Alameda, CA).

The ICG-α_v_β_6_ antibody was also mixed with venous blood of mice in Eppendorf tubes and imaged by a Nexus 128 photoacoustic instrument (Endra, Boston, MA) at laser light 710 nm and 900 nm using continuous rotation (24 s total scan time, 240 views, 1 pulse/view). The data were analyzed using Osirix software (Apple, Cupertino, CA).

For the photobleaching study, 50 μL of ICG-α_v_β_6_ antibody (1 μM) was analyzed with the photoacoustic instrument (Endra) and scanned at 710 nm and 900 nm 30 times, each scan lasting for 1 min. Photobleaching was determined by the change in photoacoustic intensity over time.

ScrIgG was also labeled in the same way to form ICG-ScrIgG as a non-targeted control.

### Binding affinity of ICG-α_v_β_6_ antibody

Competitive binding enzyme-linked immunosorbent assay (ELISA) was performed to measure the binding affinity of ICG-α_v_β_6_ antibody as described elsewhere^20^. Briefly, each well of a Costar 96-well plate (Corning, Tewksbury, MA) was coated with 0.5 μg of P2W7 antibody (anti-αv; Abcam, Cambridge, MA) at 4 °C overnight. Wells were washed with DPBS, blocked with blocking buffer (1% bovine serum albumin in DPBS) for 3 h and then washed. Fifty microliters per well of integrin α_v_β_6_ (or integrin α_v_β_3_ or integrin α_v_β_5_, 3 μg/mL; R&D Systems, Minneapolis, MN) was added for 1 h and washed with wash buffer (TBS, 0.2% Tween 20; Sigma Aldrich). Serial dilutions (1 μM–0.1 nM) of the anti-α_v_β_6_ antibody, ICG-α_v_β_6_ antibody or ICG-ScrIgG and biotinylated fibronectin (Sigma Aldrich) were incubated for 1 h and washed. Fifty microliters per well of extravidin HRP conjugate (Sigma Aldrich) was added to each well for 1 h and washed. 3,3′,5,5′-Tetramethylbenzidine (TMB; Sigma Aldrich) was then added to each well and allowed to incubate for 15 min. Absorbance at 450 nm was measured in a plate reader (Synergy Mx; BioTek, Winooski, VT).

The human squamous carcinoma cells A431 (integrin α_v_β_6_-positive)^10^ were plated at 1 × 10^4^ cells/cm^2^ in dishes and grown to ~85% confluence. ICG-α_v_β_6_ antibody or ICG-ScrIgG (0.4 μM) was added and incubated at 37 °C for 4 h. After washing with DPBS three times, fluorescence images were collected on an Eclipse Ti-E inverted microscope (Nikon, Melville, NY). Human embryonic kidney 293 T cells (integrin α_v_β_6_-negative^10^) were also incubated with ICG-α_v_β_6_ antibody in the same way. Additionally, for the blocking study, a 10-fold molar excess of unlabeled antibody was added to final concentration of 4 μM, 10 minutes before addition of ICG-α_v_β_6_ antibody into the plates of A431 cells.

### Sensitivity Study

Human squamous carcinoma cells A431 were plated at 1 × 10^4^ cells/cm^2^ in dishes and grown to ~85% confluence. The cells were incubated with 0.4 μM ICG-α_v_β_6_ antibody for 24 h at 37 °C. The cells were washed then detached with trypsin (Thermo Fisher Scientific) and centrifuged. The harvested cell pellets were re-suspended and serially diluted to 1.8, 0.9, 0.45, 0.23, 0.12, 0.06, 0.03 million in 100 μL of DPBS for PAI and fluorescence imaging.

### Small Animal Imaging and Tissue Biodistribution

All animal experiments were performed in accordance with the National Guides for the Care and Use of Laboratory Animals approved by the Institutional Animal Care and Use Committee (IACUC).

Eight-week-old female nude mice (nu/nu; Charles River Laboratories, Boston, MA) were used in this study. Two million A431 (in 100 μL DPBS) cells were injected subcutaneously in the left dorsum of the mice (n = 9). Mice bearing 0.5–0.8 cm xenografted tumors were scanned using the PAI system (Endra) at 710 nm and 900 nm using continuous rotation (24 s total scan time, 240 views, 1 pulse/view). The data were obtained before and 1 min, 1 h, 2 h, 4 h, 24 h, 48 h after administration of 200 μL of probe (2.5 μM) via tail-vein injection. The data were analyzed using Osirix software (Apple). The mice were also imaged using an optical imaging system (Bruker, Billerica, MA) for fluorescence imaging.

For *in vivo* blocking studies, a 30-fold molar excess of unlabeled anti-α_v_β_6_ antibody was intravenously injected into the mice 10 min before administration of ICG-α_v_β_6_ antibody.

Mice were sacrificed 48 h post injection of probe. The excised organs were imaged using an IVIS200 optical imaging system (Caliper Life Sciences). The data were analyzed using Living Image 4.0 software. The tumor tissue was frozen and cryosectioned at −20 °C into 5-μm slices, and fluorescence images were collected on an inverted microscope.

### Statistical Analysis

All statistical analyses were performed with SPSS 20 (IBM, Chicago, IL). The data are presented as the mean ± standard deviation. Means were compared using independent-samples *t* test. *P* values of 0.05 or less indicated significance. Nonlinear regression was performed with GraphPad Prism 5 (GraphPad, La Jolla, CA).

## Additional Information

**How to cite this article:** Zhang, C. *et al*. Photoacoustic and Fluorescence Imaging of Cutaneous Squamous Cell Carcinoma in Living Subjects Using a Probe Targeting Integrin α_v_β_6_. *Sci. Rep.* **7**, 42442; doi: 10.1038/srep42442 (2017).

**Publisher's note:** Springer Nature remains neutral with regard to jurisdictional claims in published maps and institutional affiliations.

## Supplementary Material

Supplementary Data
