# Supplementary material for: Photoacoustic and Fluorescence Imaging of Cutaneous Squamous Cell Carcinoma in Living Subjects Using a Probe Targeting Integrin αvβ6
Source: Sci Rep. 2017 Feb 9;7:42442. doi: 10.1038/srep42442 (PMC5299425; doi:10.1038/srep42442)
Supplement: Supplementary Data [file srep42442-s1.pdf]

Photoacoustic and Fluorescence Imaging of Cutaneous Squamous Cell Carcinoma in Living Subjects Using a Probe Targeting Integrin $\alpha_v\beta_6$

Chao Zhang, Yong Zhang, Kai Hong, Shu Zhu & Jie Wan

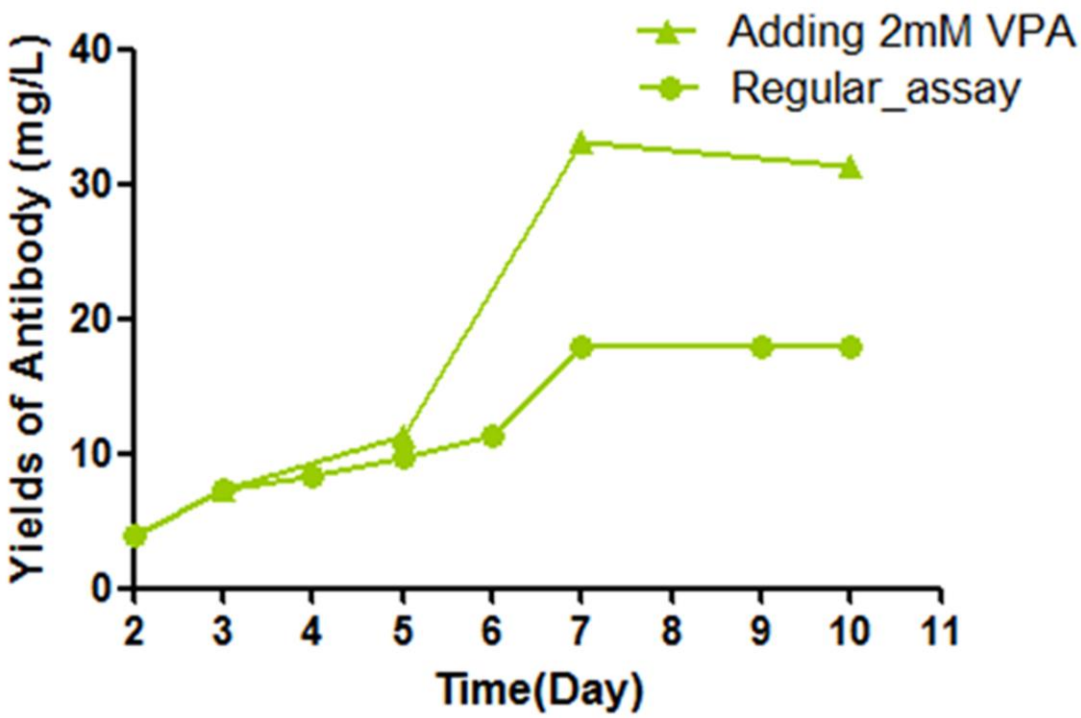

Supplementary Figure S1: Yields of antibody after days of transient co-transfection of plasmid pFUSE_H and pFUSE_L into FreeStyle 293 cells. Valproic acid (VPA) was added into expression medium 24 h after transfection to increase yields.

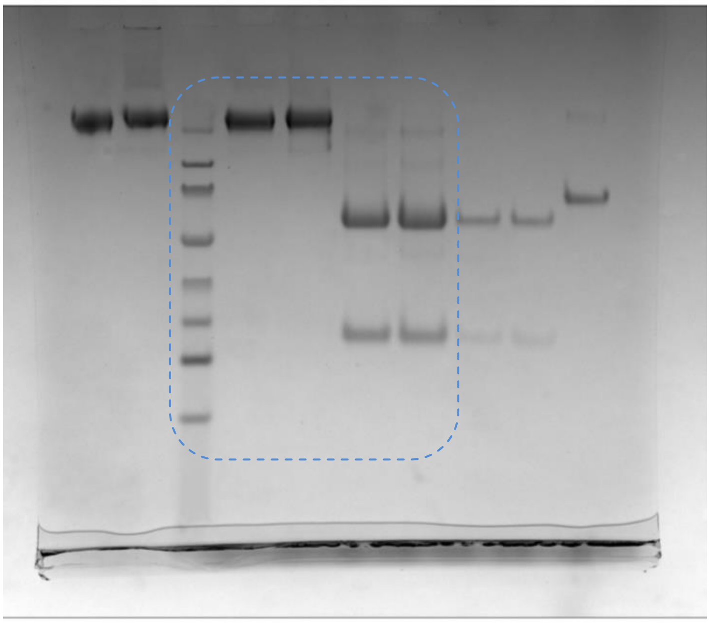

a.

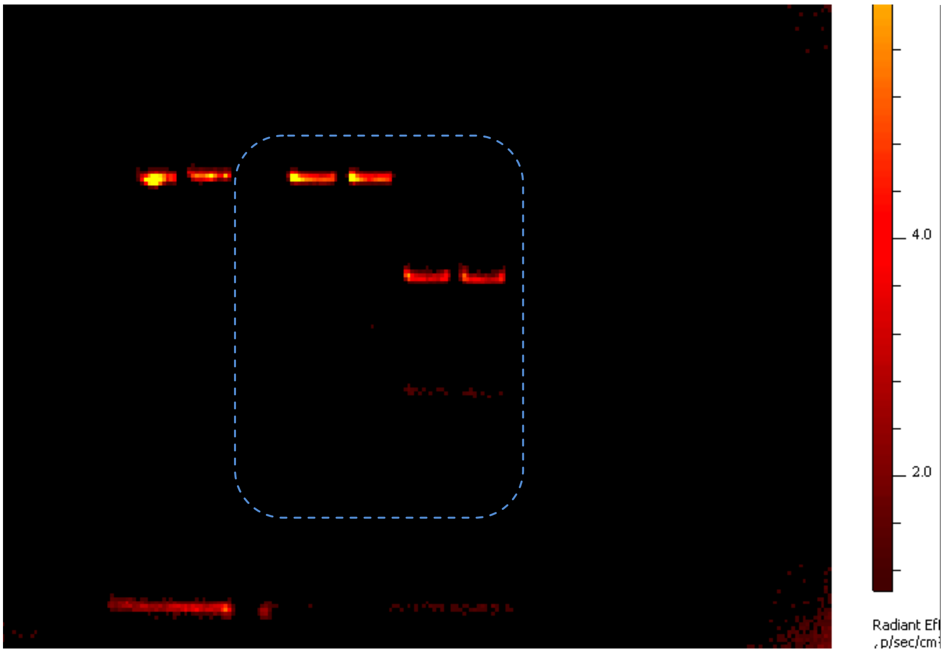

b.

Supplementary Figure S2: Full size gel of SDS-PAGE (a) and fluorescence image (b). Full length original images of the gel presented in Figure 1. The blue dotted boxes indicate cropped regions.

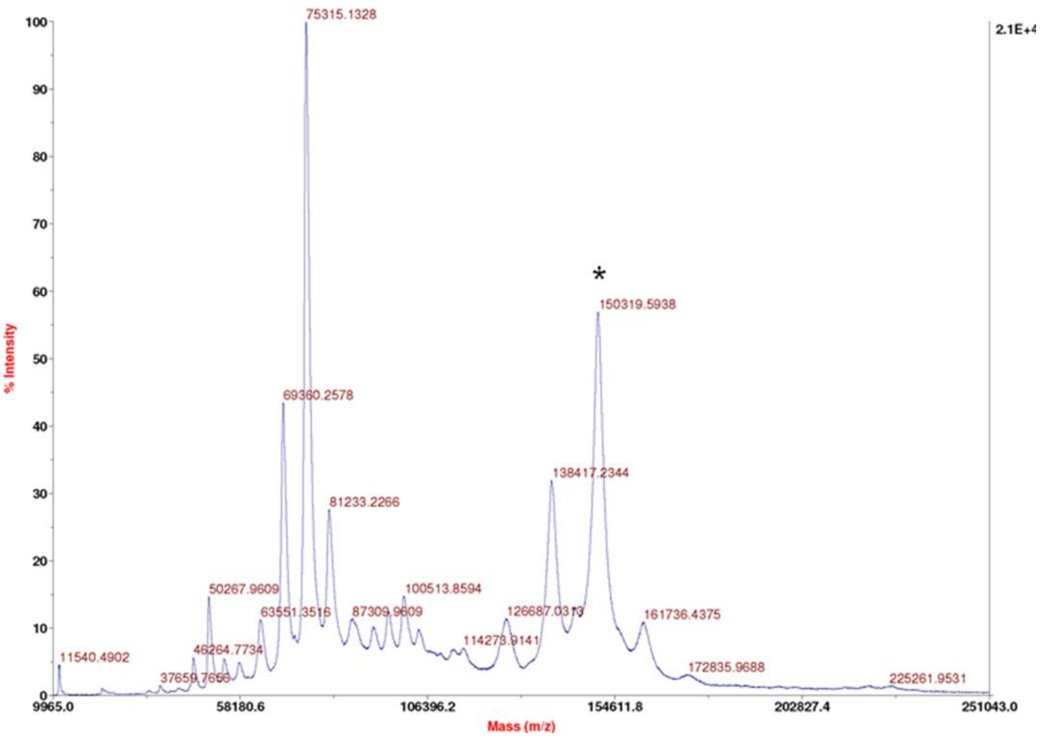

a.

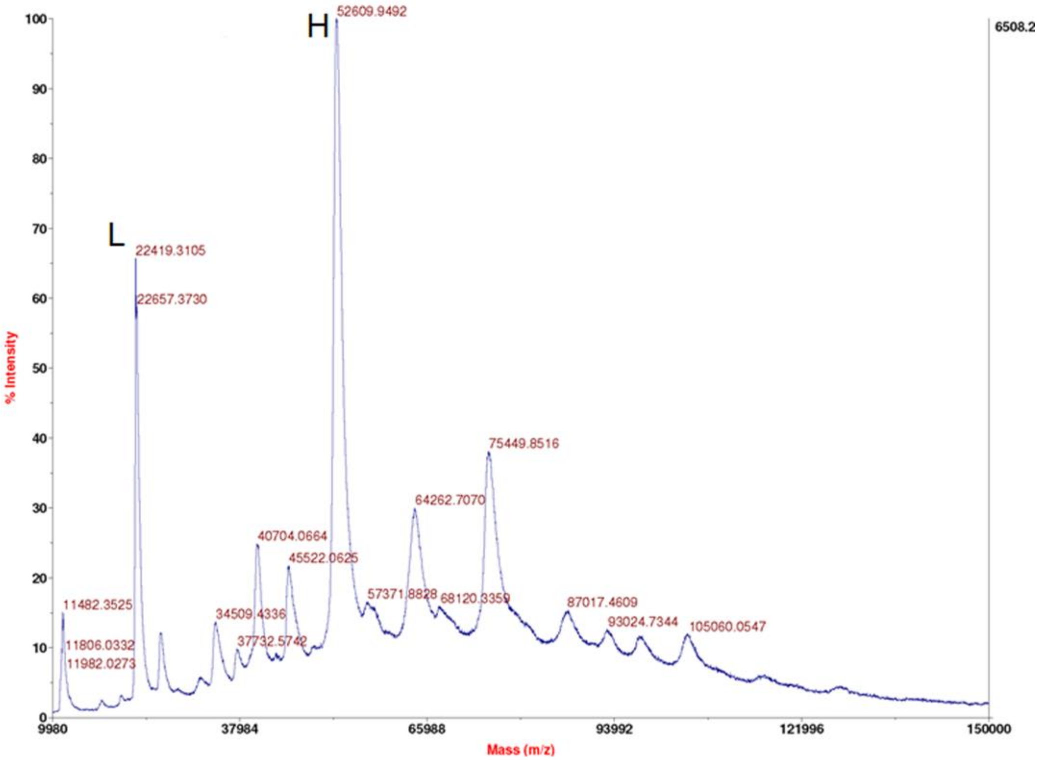

b.

Supplementary Figure S3: (a) Mass spectrum analysis of harvested antibody. Asterisk(*) shows peak of non-reduced antibody with mass of 150,319 m/z. (b) Character ‘L’ and ‘H’ show peaks of light chain and heavy chain of the reduced antibody with mass of 22,419 m/z and 52,609 m/z respectively.

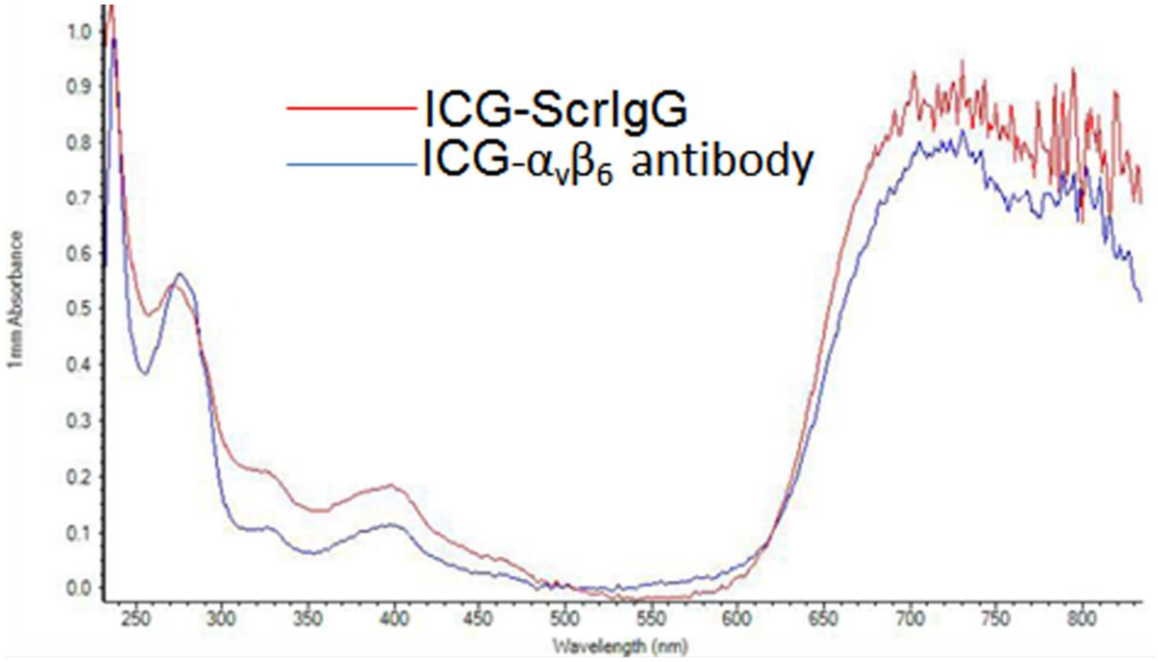

Supplementary Figure S4: Similar optical spectrum of ICG-$\alpha_v\beta_6$ antibody and ICG-ScrIgG.

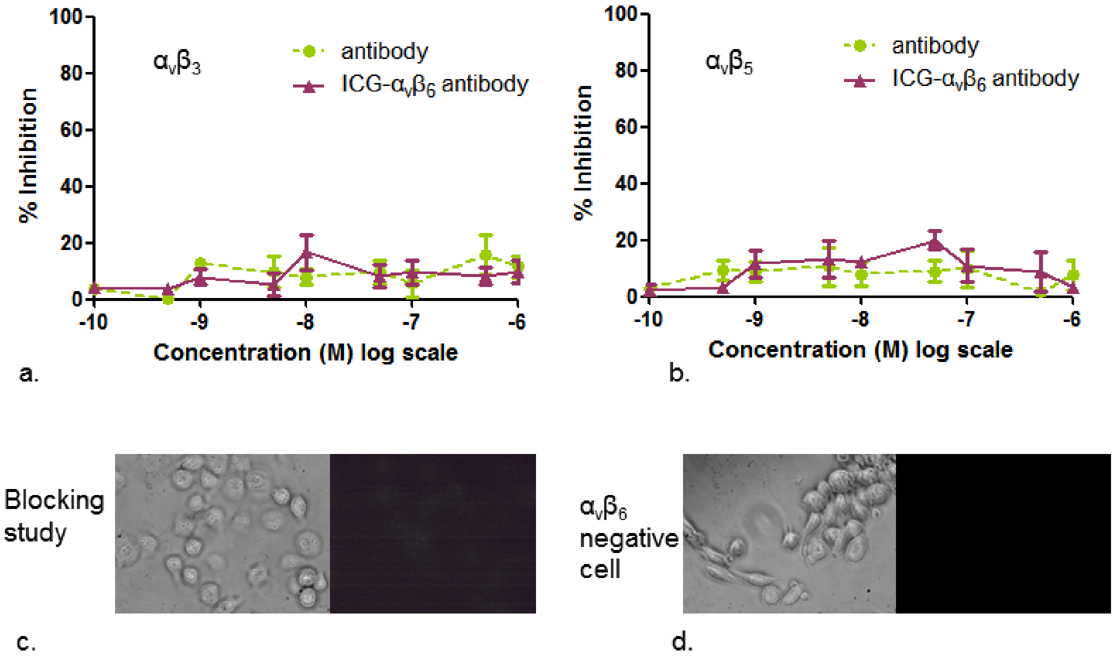

Supplementary Figure S5: Binding affinity study. Competitive binding ELISAs against integrin $\alpha_v\beta_3$ (a) and $\alpha_v\beta_5$ (b) showed no specificity of the antibody and ICG-$\alpha_v\beta_6$ antibody toward both of these two kinds of integrin. (c) Microscopic fluorescence images of cSCC A431 cells (integrin $\alpha_v\beta_6$ positive) incubated with 10 molar unlabeled antibody as blocking agent and then ICG-$\alpha_v\beta_6$ antibody for 4h at 37 ^{o}C. Limited signals were observed. (d) Microscopic fluorescence images of the human embryonic kidney 293T cells (integrin $\alpha_v\beta_6$ negative) incubated with ICG-$\alpha_v\beta_6$ antibody for 4h at 37 ^{o}C. Limited signals were observed.

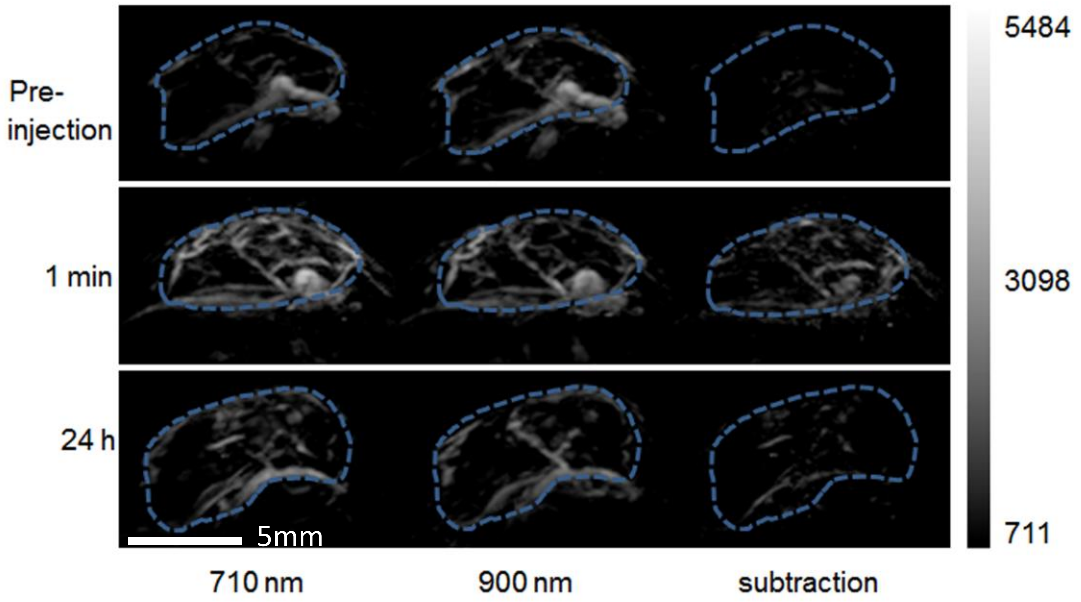
5484
Pre-
injection
3098
1 min
24 h
5mm
711
710 nm
900 nm
subtraction

a.

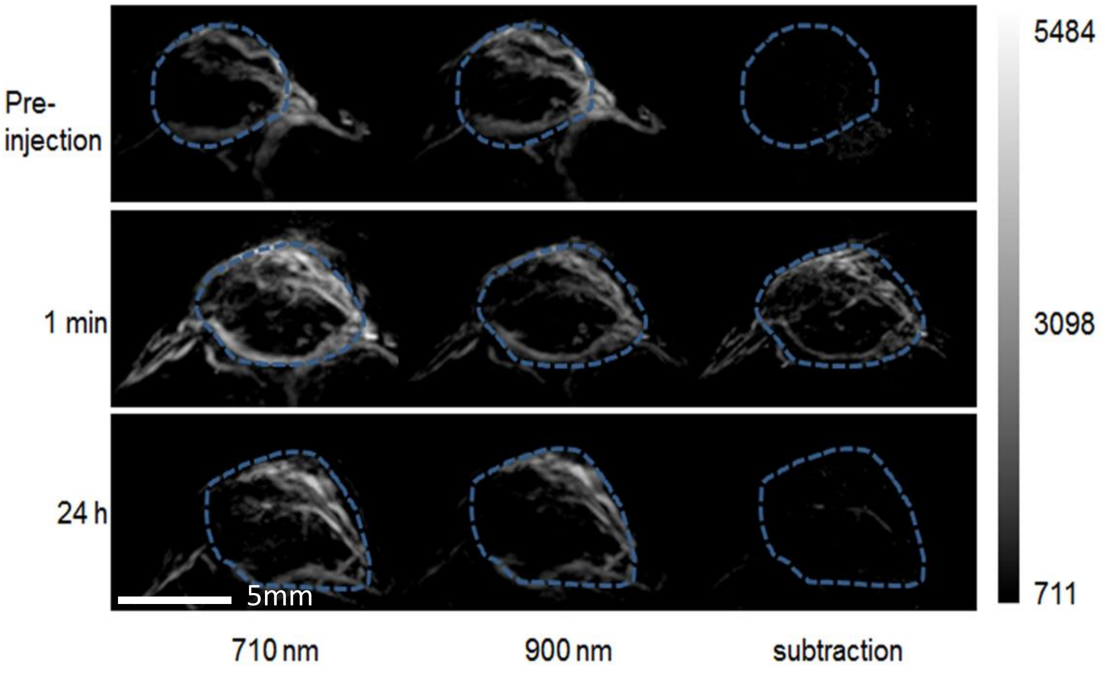
5484
Pre-
injection
3098
1 min
24 h
5mm
711
710 nm
900 nm
subtraction

b.

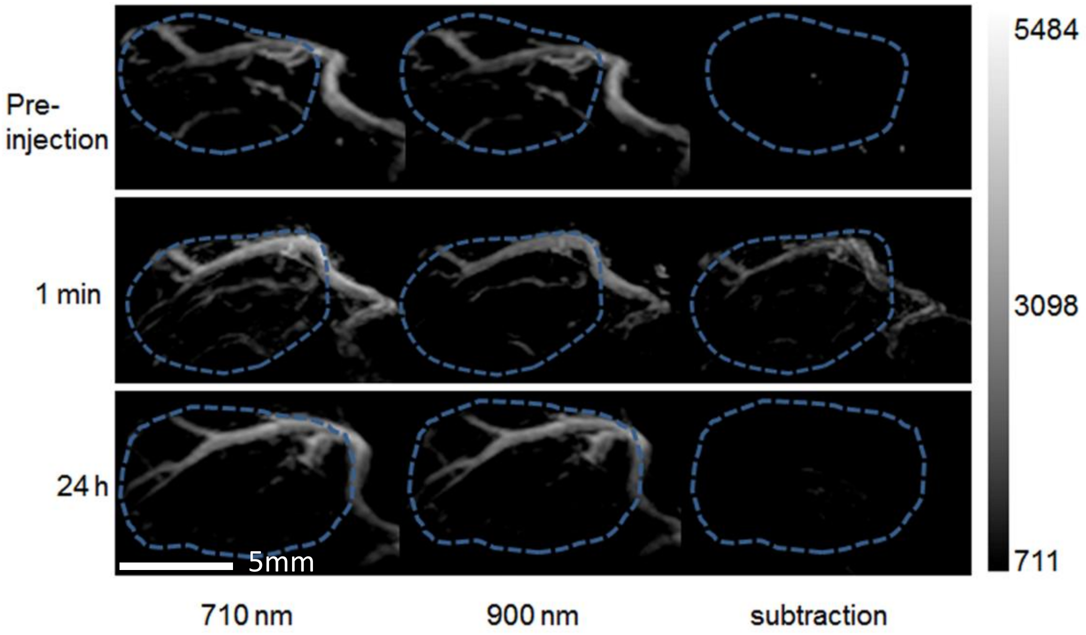

c.

Supplementary Figure S6: Photoacoustic imaging of cSCC xenograft tumors at 710 nm and 900 nm before and after injection of (a) ICG-$\alpha_v\beta_6$ antibody, (b) ICG-ScrIgG and (c) blocking with unlabeled anti-$\alpha_v\beta_6$ antibody. Photoacoustic signals of probes were unmixed by subtraction of images at 710 nm and 900 nm.

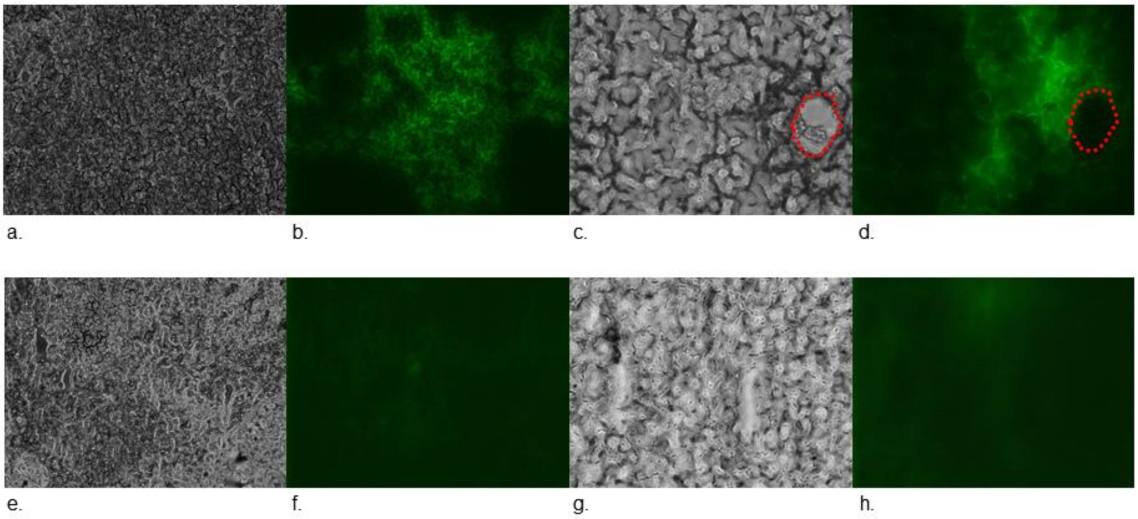

Supplementary Figure S7: Microscopic images of frozen A431 tumor tissue slices 24 h post tail vein injection of ICG-$\alpha_v\beta_6$ antibody (a-d) and ICG-ScrIgG (e-h). Magnification: 100x for figure a,b,e and f, 400x for figure c,d,g and h. Bright field for figure a, c, e and g. Fluorescence field for figure b, d, f, h. Obviously bright fluorescence could be observed on figure b and d (tail vein injection of ICG-$\alpha_v\beta_6$ antibody) than on figure f and h (tail vein injection of ICG-ScrIgG). The red circle indicates small vasculature. Brighter flurescence could be observed near the vasculature. This indicates the limited infiltration of the probe into tissues that is relatively far from vasculature.
